# Prevalence and determinants of asthma in adult male leather tannery workers in Karachi, Pakistan: A cross sectional study

**DOI:** 10.1186/1471-2458-6-292

**Published:** 2006-12-05

**Authors:** Khurram Shahzad, Saeed Akhtar, Sadia Mahmud

**Affiliations:** 1 Department of Community Health Sciences, Aga Khan University, Karachi 74800, Pakistan; 2 Family Health International, Pakistan Country Office, Islamabad, Pakistan; 3 Department of Community Medicine & Behavioral Sciences, Faculty of Medicine, Kuwait University, PO Box 24923, Safat 13110, Kuwait

## Abstract

**Background:**

This study aimed to estimate the prevalence and to identify some risk factors of adult asthma in male leather tannery workers in Karachi, Pakistan.

**Methods:**

A cross sectional study was conducted from August 2003 to March 2004 on leather tannery workers of Karachi, Pakistan. Data were collected from 641 workers engaged in 95 different tanneries in Korangi industrial area selected as sample of convenience. Face to face interviews were performed using a structured pre-tested questionnaire by trained data collectors.

**Results:**

Prevalence of adult asthma was 10.8% (69/641) in this study population. The prevalence of perceived work-related asthma was 5.3% (34/641). Multivariable logistic regression model showed that after taking into account the age effect, the leather tannery worker were more likely to be asthmatic, if they were illiterate (adjusted OR = 2.13, 95% CI: 1.17–3.88), of Pathan ethnicity (adjusted OR = 2.69; 95% CI: 1.35–5.36), ever-smoked (adjusted OR = 2.22, 95% CI: 1.16–4.26), reportedly never used gloves during different tanning tasks (OR = 3.28; 95% CI : 1.72–6.26). Also, the final model showed a significant interaction between perceived allergy and duration of work. Those who perceived to have allergy were more likely to have asthma if their duration of work was 8 years (adjusted OR = 2.26; 95% CI: 1.19 – 4.29) and this relationship was even stronger if duration was 13 years (adjusted OR = 3.67; 95% CI: 1.98–6.79).

**Conclusion:**

Prevalence of asthma in leather tannery workers appears to be high and is associated with educational status, ethnicity, smoking, glove use, perceived to have allergy and duration of work.

## Background

Asthma is one of the most common chronic inflammatory disease of airways [1], characterized by hyper responsiveness to a variety of stimuli. Among the various risk factors of asthma, those of occupational origin are gaining more importance with time due to use of various chemicals in industries which potentially induce hypersensitivity and predisposition to asthma [2,3].

Leather tanning is principally chemical preservation of raw hide by a process in which binding of various chemicals (mainly chromium salts e.g. potassium dichromate) to proteins in raw hide takes place. Chromium has potential to bind with skin proteins of tannery workers to produce complex antigens which lead to hypersensitivity. The resulting contact dermatitis could be preliminary condition to the development of bronchial asthma [3]. Tannery workers are thus potentially exposed to harmful agents rendering them vulnerable to health problems especially those of respiratory tract and skin.

There is paucity of published data on the magnitude of asthma among leather tannery workers. Varying prevalence of asthma (2.2% and 38%) among leather tannery workers in India has been reported previously [4,5], with moderate to high exposures at workplace to be significantly associated with asthma [5]. There is need to further study prevalence and risk factors of asthma in tannery workers to better account for asthma burden and to identify high risk exposures in factory environment for prevention purposes. To our knowledge no published data are available on prevalence and risk factors of asthma in tannery workers in Pakistan. This study aimed to estimate prevalence of asthma and to identify some risk factors associated with it in adult male leather tannery workers of Karachi, Pakistan

## Methods

### Study design, setting and population

This cross sectional study was conducted from August 2003 to March 2004 among male leather tannery workers of Korangi industrial area of Karachi. Target population was male leather tannery workers of Karachi. Only those workers were enrolled who were working with tanning process (i.e. handling from raw hide to finished leather). Workers from administrative and other departments of tannery were excluded. Most of the tanneries in Karachi are located in Korangi industrial area. There are total 95 tanneries in Korangi Industrial Area.

### Sampling design and strategy

All (95) factories in Korangi industrial area were contacted by mail or phone. Thirteen factories allowed their workers to be interviewed during factory timing. For other factories team visited the colonies where workers lived and interviewed them there. Almost 40% to 50% of the workers associated with tanning process were selected from each tannery as a sample of convenience. Finally, 641 workers employed in 95 different factories were interviewed.

### Definitions

In this study, a subject was labeled as asthmatic if he met one or more of the following criteria.

a) Diagnosed asthmatic by a physician or a nurse [6-8].

b) Having the following symptoms.

i. Attacks of shortness of breath with wheezing in the past 12 months [9].

ii. Normal breathing in between episodes of shortness of breath.

A worker reporting improvement of symptoms on going to leave or weekend and/or worsening of symptoms on coming back from short leave was labeled as having work-related asthma [10]. Perceived to have allergy was defined as self perception of allergy to at least one of the substances i.e. food, metals, chemicals, medicine, dusts or animals.

### Data collection

A team of three experienced data collectors were trained in conducting interviews. For the interviews conducted in factories, planned visits were done. A quite and clean room was taken from administration and workers were requested to come to the room. In case of home interviews team took appointments from workers a day before and then visited houses at scheduled timings. Study subjects were briefed about the study. Confidentiality was ensured to every worker and approximate time of form filling was informed. Verbal informed consent was taken. The study was approved by the Institutional Ethical Review Committee.

### Statistical analysis

Descriptive analysis was done to describe characteristics of our study population. Univariate odds ratios (OR) and their 95% confidence interval (CI) were used to screen predictors of asthma. Multiple logistic regression analysis was performed to evaluate independent effect of variables selected from univariate analysis (*p *≤ 0.15). Variables which were either not significant or non-confounders were eventually removed. Scale of continuous variables was assessed using quartile analysis and Box-Tidwell transformation. After developing main effect model we evaluated all the biologically meaningful interactions between independent variables. Interactions that were significant at 10 % level were included in the model. The final model was assessed for goodness of fit using Hosmer and Lemeshow test [11]. All analyses were done using Statistical Package for Social Sciences (SPSS) version 10.0.

## Results

Table 1 shows the descriptive characteristics of 641 subjects included in this study. Mean (± SD) age (years) of respondents was 27 (± 9) with a median of 25. There were two major ethnic groups i.e. Punjabi (53%) and Pathan (33%). Other ethnic groups composed of Urdu, Balochi, Sindhi and Brahvi. Most of the workers were employed on daily wages (77%). The mean (± SD) income (rupees per month; One US $ = 60.25 Pak rupees) was 4308 (± 2233) and median income was 4000 rupees per month. Income ranged from 1,000 to 55,000 rupees per month and almost 49% of the workers had income between 3000 to 5000 rupees per month. Approximately 41% of the workers were illiterate, about 20% had education between 1–5 years and 35% between 6–10 years. Majority of workers were living in rented houses (59%). The mean (± SD) duration of work (years) was 8 (± 6.8), and median duration was 6 years.

Of 641 workers included in the study, 69 (10.8%) subjects reportedly were asthmatic. Of 69 asthmatic subjects, 34 (49%) reported an association of their symptoms with work. Univariate analyses showed that age, educational status, ethnicity, smoking, glove use, perceived to have allergy, *naswar *use (naswar is a mixture of tobacco, chemicals and some herbs usually kept in oral cavity between teeth and lips) and duration of work were associated with asthma (Table 2).

Final multivariable logistic model included age, educational status, ethnicity, smoking status, glove use and an interaction between perceived allergy and duration of work (Table 3). The effect of allergy was evaluated at mean duration of work (8 years); also the effect of allergy with 5 additional years of work duration (13 years of work duration) was evaluated. Illiterate were more likely to have asthma compared to those who were literate (adjusted OR = 2.13; 95% CI: 1.17–3.88). Pathan workers were more likely to have asthma compared to workers of Punjabi ethnicity (adjusted OR = 2.69 95% CI: 1.35–5.36). The workers who did not report the use of gloves were more likely to have asthma compared to those who at least rarely used them during different tanning tasks (adjusted OR = 3.28; 95% CI: 1.72–6.26). Ever smokers were more likely to have asthma compared to those who had never smoked (adjusted OR = 2.22; 95% CI: 1.16–4.26). As noted earlier, final model included a significant interaction (*p *= 0.02) between perceived allergy and duration of work in tannery. The workers who perceived to have allergy, were more likely to have asthma if their duration of work was 8 years (adjusted OR = 2.26; 95% CI: 1.19 – 4.29) and this relationship was even stronger if duration was 13 years (adjusted OR = 3.67; 95% CI: 1.98–6.79). However, there was non-significant relationship between duration of work and asthma for those who did not have perceived allergy.

## Discussion

The questionnaire used in this study has been validated elsewhere [9]. The validity of questionnaire in the context of our population is not known. The question about "physician diagnosed asthma" has a high specificity (99%). Respiratory symptoms, on the other hand, have a lower specificity. Specificity of wheeze has been reported to range from 66 to 96% in different European countries [9].

This cross sectional study estimated an asthma prevalence of 10.8% in leather tannery workers of Karachi and to our knowledge is the first report from Pakistan. Study also showed that ethnicity, educational status, smoking, perceived allergy and glove use were significantly associated with asthma in this study population. To our knowledge only two studies have estimated asthma prevalence in tannery workers. Shukla et al. found a prevalence of 2.2% for occupational asthma in tannery workers in India [4]. Physician examination was used to diagnose asthma but no objective tests were done to establish occupational etiology of asthma. Ory and colleagues estimated an asthma prevalence of 38% in tannery workers in a study conducted in Kanpur, India [5]. Lower prevalence found by Shukla et al. is due to greater emphasis on clinical findings during medical examination. Response rate was also lower in their study. Ory and colleagues used more sensitive criteria and developed a close co-operation among the project team, tannery owners and tannery workers. The difference in prevalence found in this study (10.8%) and that reported by Ory et al. (38%) might be due to a different set of criteria used for diagnosing asthma. On the other hand asthma prevalence in our study population is higher compared to some of the general populations, including those of South-east Asia. It is higher than that reported in men in Iran (2.4%) [12], Bangladesh (4.4%) [13] and India (4%) [14]. In the local context, the lack of data on asthma prevalence in general population in Pakistan precluded possible comparison with asthma prevalence in our study population.

In this study Pathans were at increased risk of having asthma compared to Punjabis. The effect of ethnicity on asthma in our population could be due to certain socio-economic and behavioral differences among the races. The possible role of illiteracy seems to be limited due to the fact that its effect was taken into account in multivariable analysis in this study. The association could still be confounded by certain other un-measured socioeconomic and behavioral factors in this study. However, a possibility of underlying genetic causation cannot be excluded as demonstrated previously [15,16]. Race was found to be a significant predictor of asthma in a study done in United States, which found Caucasians and Latinos to be at greater risk for asthma compared to African American [16]. A case control study showed that eastern European and Scandinavians were more likely to develop asthma compared to British and other ethnic groups [17].

Illiterate were more at risk of having asthma in this study. A study conducted in Bangladesh in general population has also revealed that illiterate were at greater risk of asthma [13]. Another study has also shown education to be protective for asthma. This study was twin matched case-control study, and thus adjusted for most of the childhood factors such as diet, allergen exposure, passive smoking, parental characteristics and genetic factors [18]. Mechanism by which education may have its protective effect against asthma might be by improved general health and well being of the subject and/or by increased awareness about triggering factors, subsequently avoidance of exposure to such factors.

Glove use was protective for asthma in our population. Liss et al. have observed the effect of glove use on asthma in different tasks among radiographers. Latex glove use was found to be protective for two or more work-related respiratory symptoms [19]. Latex glove use, on the other hand, has been known to induce asthma and hypersensitivity due to their latex content [20,21]. We did not ascertain the material of gloves and thus it was not possible to comment on this effect. Protective effect for asthma in our population might be due to protection of skin from harmful chemicals which are so well known to cause hypersensitivity and allergy [2,22]. Skin exposure to chemicals have been shown to play a role in initial immunologic sensitization [23], which is a very important link in causal chain for asthma. By decreasing the likelihood of allergy and hypersensitivity to these chemicals by use of gloves would potentially prevent bronchial hyper responsiveness and asthma on exposure to their fumes and dusts.

Smoking was a significant risk factor for asthma in our population. Both previous studies on tannery workers have not evaluated the effect of smoking on asthma. Studies done recently in general populations have shown a positive association between smoking and asthma [13,19,24,25]. Current and former smokers have been found to have increased risk of asthma in a recent study conducted in United States [25]. A study done in Korean population showed active smoking to be risk factor for asthma. No significant difference between past and current smokers in terms of asthma was observed in this study [24]. In a study conducted in Turkey, smoking was found to be a risk factor in occupational asthma in car and furniture painters [26]. Few studies have shown asthma to be independent of smoking [27,28]. In work settings at least one study has found smoking to be protective for asthma for work in the current industry [7]. Protective effect is possibly due to the self selection effect i.e. those subjects who already have sensitive airways and are prone to asthma are less likely to start smoking.

In the final model there was significant interaction between duration of work in leather tanning job and perceived allergy to food, metals, chemicals, medicine, dusts and animals. Those who reported to have perceived allergy were at excess risk of asthma with increasing duration of work in tanning compared to risk estimates of perceived allergy and duration of work separately. Exposures to allergens thus seem to have a cumulative effect for those who are already allergic to above mentioned substances. Duration of work, however, was not a risk factor for a worker who didn't report perceived allergy to above noted substances. This further strengthens evidence that allergy acts as a predisposing factor for development of asthma. The ability of chromium binding with skin proteins may produce complex antigen leading to hypersensitivity [29]. This finding also shows that occupational factors may play a synergistic role with pre-existing predisposition for allergy. Meer et al. reported similar findings in general population in Netherlands and showed an excess risk of asthma if atopy and organic dust exposure occurred concurrently [30]. The number of workers who underwent pre-placement and regular physical examination in our study was low. Therefore, the possibility of workers exclusion from the profession on being positive on skin antigen testing or on having signs and symptoms of a respiratory illness is also low.

This study has some limitations. Diagnosis of asthma was based on either physician diagnosis or asthmatic symptoms in past 12 months. This method of diagnosis could inflate asthma cases due to similar presentation of diseases like COPD [31,32]. However, we did make an effort to minimize misclassification by excluding subjects which reported a continuous breathing problem rather then intermittent [33]. Asthma, on the other hand, could be under-reported due to lower sensitivity of questions on wheezing and breathlessness. In addition, risk estimates are also likely to be diluted if the specificity of the questions is low [9]. For population based epidemiologic studies, there is no satisfactory definition of asthma. Comparison of results of different studies is difficult because of the use of different protocols and instruments [34]. Such difficulty in defining asthma has also been encountered in studies done elsewhere [13,35,36].

Healthy worker effect may have occurred in our population (as in other occupational epidemiologic studies). The workers who associated their asthma symptoms to work might have left the job at a higher rate compared to those who either did not have asthma or did not associate the aggravation of their asthma to work [37]. In an attempt to estimate and adjust for healthy worker effect we tried to get access to industries data, to get information of number of subjects who have left job or retired during past three years and their main reason(s) of retirement. However, access to such data was denied by industry owners. Selection of healthy workers from baseline population [38] is likely to be less important in our study population as proportion of those having pre-placement examination was low, and it is not a usual practice in our setting to screen workers for allergy and to exclude those workers from occupation, who are more at risk of developing certain conditions. Prevalence of asthma is likely to have been underestimated in this study due to this healthy workers effect.

There may also have been a differential recall of allergic episodes between asthmatic and non-asthmatic workers, with asthmatic subjects recalling the allergic episodes more easily than non-asthmatics. This might have led to overestimation of risk related to allergy.

Study subjects were included as sample of convenience. Therefore, generalization of our results may be seen only in the context of study population rather than the target population of workers of leather tanning in Karachi. However, non-response to participate in the study was nearly nil, since only one worker left with incomplete interview.

## Conclusion

This study showed a high prevalence of asthma in tannery workers. Prevalence is higher compared to adult males in general population of some of the countries. However comparable data on prevalence of asthma in general population in Pakistan were not available. This study showed that educational status, ethnicity, smoking status, perceived allergy and glove use are significantly associated with asthma. Further research needs to be done on asthma in general population and tannery workers in Pakistan, with use of objective tests to demonstrate occupational nature of the diseases.

## Competing interests

The author(s) declare that they have no competing interests.

## Authors' contributions

KS developed research protocol, hired and trained research team, supervised data collection, did data analysis and report writing. SA provided major inputs in proposal development and refinement, interpretation of results of data analysis and had major contribution in report writing. SM provided inputs in research tool, data analysis, and interpretation and contributed to report writing.

## Pre-publication history

The pre-publication history for this paper can be accessed here:



## Figures and Tables

**Table 1:** Descriptive characteristics of leather tannery workers of Karachi, Pakistan from August 2003 to March 2004 (n = 641*)

**Variables**	**n (%)**
Marital status	
Currently married	329 (51.3)
Currently unmarried	312 (48.7)
Completed years of formal education	
0 years	264 (41.2)
1 – 5 years	131 (20.4)
6 – 10 years	224 (34.9)
11 years or more	22 (3.4)
Monthly income	
Did not reveal	30 (4.7)
1000–3000	206 (32.1)
3001–5000	312 (48.7)
5001+	93 (14.5)
Type of job contract	
Daily wages	491 (77.1)
Permanent	146 (22.9)

* The total for some of the variables does not add up to 641 due to missing values

**Table 2:** Univariate analysis of factors associated with asthma among leather tannery workers, Karachi, Pakistan, August 2003 to March 2004 (n = 641^†^)

**Variables**	**n**	**OR**	**95%CI**	***p***
Age	641	1.06	1.02, 1.08	<0.01
Educational status				
Literate	377	1.00	-	
Illiterate	264	2.45	1.47, 4.09	<0.01
Type of house				
Pacca	582	1.00		
Katcha	57	0.78	0.30, 2.02	0.61
Ethnicity				
Punjabi	334	1.00	-	<0.01
Pathan	211	3.32	1.91, 5.76	
Others	91	1.18	0.49, 2.86	
Place of interview				
Factory	194	1.00		
Outside factory	416	2.30	1.21, 4.42	0.01
House ownership				
Owned	262	1.00	-	
Rented	376	0.96	0.58, 1.59	0.96
Smoking status				
Non-smoker	455	1.00	-	
Ever smoker	182	2.11	1.26, 3.51	0.04
Naswar use^‡^				
No	150	1.00	-	
Yes	486	2.47	1.47, 4.15	0.01
Perceived allergy^§^				
No	152	1.00	-	
Yes	475	3.71	2.20, 6.27	<0.01
Working hours per day	641	1.01	0.98, 1.05	0.47
Exposure to chemicals				
No	56	1.00	-	
Yes	583	0.69	0.31, 1.52	0.36
Knowledge about hazards of chemicals				
No	33	1.00	-	
Yes	543	1.15	0.39, 3.40	0.80
Glove Use				
User**	268	1.00	-	
Non-user^††^	373	2.93	1.25, 4.54	<0.01
Total duration of work	641	1.08	1.04, 1.12	<0.01
Exhaust fan use				
No	127	1.00	-	
Yes	355	0.91	0.47, 1.75	0.78
Air sucker use^‡‡^				
No	367	1.00	-	
Yes	115	0.68	0.32, 1.43	0.31
Water sucker use^§§^				
No	411	1.00	-	
Yes	71	1.12	0.50, 2.49	0.41
Provision of gloves				
No	122	1.00	-	
Yes	447	0.78	0.42, 1.42	0.42
Provision of masks				
No	167	1.00	-	
Yes	402	0.88	0.51, 1.55	0.66
Coverage for health***				
No	437	1.00	-	
Yes	131	0.87	0.47, 1.58	0.64
Pre-placement and regular physical examination				
No	415	1.00	-	
Yes	80	0.76	0.38, 1.55	0.56

^† ^Total for some of the variables does not add up to 641 due to non-response

^‡ ^Naswar is a mixture of tobacco, chemicals and some herbs usually kept in oral cavity between teeth and lips

^§ ^Perceived allergy to at least one of the following substances: food, metals, chemicals, medicine, dusts or animals

** worker who has at least rarely used gloves during different tanning tasks

^†† ^Worker who has never used gloves

^‡‡ ^An instrument placed close to a work station to drain away polluted air in order to keep a low concentration of pollutants at worksite

^§§ ^An instrument placed close to a workstation producing waste water. It prevents logging and accumulation of harmful waste water

*** Whether workers are offered reimbursements against their health care expenses

**Table 3:** Multiple logistic regression analysis of factors associated with asthma among leather tannery workers, Karachi, Pakistan, August 2003 to March 2004 (n = 620^†††^)

**Variables**	**adjusted OR^‡‡‡^**	**95% CI**
Educational Status		
Literate	1.00	-
Illiterate	2.13	1.17, 3.88
Ethnicity		
Punjabi	1.00	-
Pathan	2.69	1.35, 5.36
Others^§§§^	1.70	0.63, 4.58
Smoking status		
Non-smoker	1.00	-
Ever smoker	2.22	1.16, 4.26
Glove use		
User****	1.00	-
Non-user^††††^	3.28	1.72, 6.26
Allergy^‡‡‡‡ ^and duration of work^§§§§^		
Non-allergic		
8 years duration of work	1.00	-
13 years duration of work	0.99	0.73, 1.33
Allergic		
8 years duration of work	2.26	1.19, 4.29
13 years duration of work	3.67	1.98, 6.79

^†††^The final model included less than 641 subjects due to missing values

^‡‡‡ ^All variables are mutually adjusted for each other

^§§§ ^Other ethnicity includes Urdu, Sindhi, Balochi and Brahvi

*** Worker who has at least rarely used gloves during different tanning tasks

^†††† ^Worker who has never used gloves

^‡‡‡‡ ^Perceived allergy to at least one of the following substances: food, metals, chemicals, medicine, dusts or animals

^§§§§ ^The interaction was analyzed by calculating odds ratio relative to a common reference i.e. the duration of work at mean duration of 8 years and non-allergic status
